# Testicular Heat-Shock Protein Expression in Rats Following 3.5 GHz and 24 GHz RF-EMF Exposure

**DOI:** 10.3390/ijms27083452

**Published:** 2026-04-12

**Authors:** Syed Muhamad Asyraf Syed Taha, Farah Hanan Fathihah Jaffar, Atikah Hairulazam, Sivasatyan Vijay, Norazurashima Jamaludin, Aini Farzana Zulkefli, Mohd Farisyam Mat Ros, Khairul Osman, Zahriladha Zakaria, Mohd Amyrul Azuan Mohd Bahar, Siti Fatimah Ibrahim

**Affiliations:** 1 Department of Physiology, Faculty of Medicine, Universiti Kebangsaan Malaysia (UKM), Jalan Yaacob Latif, Bandar Tun Razak, Cheras, Kuala Lumpur 56000, Federal Territory, Malaysia; p126219@siswa.ukm.edu.my (S.M.A.S.T.); p129544@siswa.edu.ukm.my (A.H.); p133758@siswa.ukm.edu.my (S.V.); ainifarzana@hctm.ukm.edu.my (A.F.Z.); mdfarisyam@ukm.edu.my (M.F.M.R.); 2 Forensic Science Program, Center for Diagnostic, Therapeutic and Investigative Studies (CODTIS), Faculty of Health Sciences, Universiti Kebangsaan Malaysia (UKM), Jalan Raja Muda Abdul Aziz, Kuala Lumpur 50300, Federal Territory, Malaysia; norazurashimajamaludin@gmail.com (N.J.); khairos@ukm.edu.my (K.O.); 3 Faculty of Electronics & Computer Technology and Engineering, Universiti Teknikal Malaysia (UTeM), Hang Tuah Jaya, Durian Tunggal 76100, Melaka, Malaysia; zahriladha@gmail.com; 4 Intel Microelectronics (M) Sdn. Bhd., Bayan Lepas Technoplex, Medan Bayan Lepas, Bayan Lepas 11900, Pulau Pinang, Malaysia; amyrul.azuan.mohd.bahar@intel.com

**Keywords:** electromagnetic fields, heat shock protein, reproductive health, Western blotting, non-ionizing

## Abstract

The expansion of fifth-generation (5G) wireless networks has increased environmental exposure to mid-band and millimeter-wave radiofrequency electromagnetic fields (RF-EMF), but their molecular effects on male reproductive tissues remain insufficiently understood. This study evaluated whether repeated exposure to 3.5 GHz and 24 GHz RF-EMF alters testicular stress-associated molecular responses by integrating electromagnetic dosimetry with an in vivo rat model. Whole-body specific absorption rate (SAR) and 10 g peak SAR were estimated using a rat voxel model and scaled to the 20 cm antenna-to-cage geometry used during exposure. Thirty-six adult male Sprague Dawley rats were allocated to sham, 3.5 GHz, or 24 GHz groups and exposed for 1 h/day or 7 h/day over 60 days. Testes were examined histologically and assessed for HSP27, HSP70, and HSP90 protein expression. SAR values were low overall, although absorption was higher at 3.5 GHz than at 24 GHz. Histological evaluation showed preserved seminiferous tubule architecture without consistent structural injury. In contrast, molecular analysis demonstrated frequency- and duration-dependent modulation of heat shock proteins, including early HSP70 downregulation at both frequencies, followed by HSP90 upregulation at 3.5 GHz and HSP27 upregulation at 24 GHz. These findings indicate that low-level 5G-relevant RF-EMF exposure can modify molecular stress responses in testicular tissue even in the absence of overt histological damage.

## 1. Introduction

Fifth-generation (5G) networks use both mid-band frequencies around 3.5 GHz and millimeter-wave (mmWave) bands, such as 24 to 28 GHz, to increase capacity and data rates relative to previous generations [1]. International exposure guidelines for radiofrequency fields in the 100 kHz to 300 GHz range, which include 5G frequencies, are intended to prevent established adverse health effects by limiting whole-body and local exposure [2]. Available compliance assessments indicate that typical environmental 5G exposures remain well below these limits [1,3]. Nevertheless, concern persists regarding male reproductive health, because some experimental and epidemiological studies at earlier RF frequencies have reported alterations in semen-related and testicular endpoints, although the overall evidence remains inconsistent [4].

Interpretation of RF-EMF bioeffects depends on well-defined dosimetry. In the sub-6 GHz range, exposure is commonly quantified using specific absorption rate (SAR, W kg^−1^), which reflects volumetric energy absorption within tissue. At higher frequencies, particularly above 6 GHz, energy deposition becomes increasingly superficial, and contemporary exposure standards therefore express local basic restrictions primarily in terms of absorbed power density rather than SAR [2]. Nevertheless, computational dosimetry studies frequently report SAR alongside power density metrics to support cross-frequency comparisons and to characterize whole-body absorption in anatomically realistic models, particularly under near-field exposure conditions where incident power density alone may be insufficient [5]. Because 3.5 GHz and 24 GHz differ substantially in penetration depth and spatial energy deposition, they may not induce identical biological responses in reproductive tissues [5].

The testis is highly sensitive to temperature change and other environmental insults and has therefore been proposed as a potential target of RF-EMF effects [6]. Experimental studies in rodents exposed to pre-5G frequencies, particularly around 900 to 2100 MHz, have reported changes in oxidative stress, apoptosis-related pathways, and testicular protein expression [6,7,8,9]. However, these effects are often modest and appear to depend on dosimetry quality, temperature control, and cumulative exposure pattern, with overt histological injury frequently minimal or absent [4]. In vivo studies addressing the specific mid-band and mmWave frequencies used in 5G networks under exposure conditions characterized by contemporary dosimetric approaches remain limited [1,3,5].

Heat shock proteins (HSPs) are stress-responsive molecular chaperones that play central roles in proteostasis, cell survival, and apoptotic regulation [10]. In the testis, HSP70 and HSP90 contribute to spermatogenesis through cytoprotective and regulatory functions, while HSP27 has been associated with stress adaptation in both somatic and germ cell populations [10,11,12]. These proteins are therefore plausible molecular indicators of subtle RF-EMF-induced stress that may precede overt structural damage in reproductive tissue [10].

Only a limited number of in vivo studies have examined testicular HSP responses after RF-EMF exposure, including *HspA2* upregulation after 2100 MHz exposure in mice and HSP70 immunoblot changes after combined CDMA and WCDMA exposure in rats [13,14]. To our knowledge, no in vivo animal study has compared testicular HSP27, HSP70, and HSP90 responses after repeated exposure to 3.5 GHz and 24 GHz under low-level conditions defined by numerical dosimetry and realistic source-to-animal separation. Therefore, this study investigated whether prolonged exposure to 3.5 GHz and 24 GHz RF-EMF alters stress-associated molecular responses in the testes of Sprague Dawley rats, with histology included as tissue-level context. Specifically, testicular morphology and HSP27, HSP70, and HSP90 expression were assessed after 60 days of daily 1 h or 7 h exposure at a 20 cm source-to-animal distance. However, direct dosimetric assessment at 20 cm was not performed in the current study; the 20 cm exposure values used in the animal study were estimated from the 5 cm and 10 cm simulations using local power-law extrapolation. We hypothesized that both frequencies would modify testicular HSP expression, but that the response pattern would differ by frequency because of their distinct dosimetric characteristics. Given these differences, the interpretation of RF-EMF bioeffects requires careful consideration of possible thermal contributions.

## 2. Results

### 2.1. Estimation of SAR

Table 1 and Table 2 summarize the normalized Max SAR_10g_ and Total SAR values obtained from the 3.5 GHz and 24 GHz simulations. At 5 cm, Max SAR_10g_ was higher at 24 GHz than at 3.5 GHz. At 10 cm, this pattern reversed, with higher Max SAR_10g_ at 3.5 GHz than at 24 GHz. Total SAR was similar between frequencies at 5 cm, but at 10 cm, it was higher at 3.5 GHz than at 24 GHz. For both metrics, values decreased with increasing antenna-to-model distance. At the extrapolated 20 cm condition, both Max SAR_10g_ and Total SAR were lowest for both frequencies. Under the extrapolated 20 cm condition, both metrics were estimated to be higher at 3.5 GHz than at 24 GHz.

### 2.2. Testicular Histological Changes

**Figure 1:**
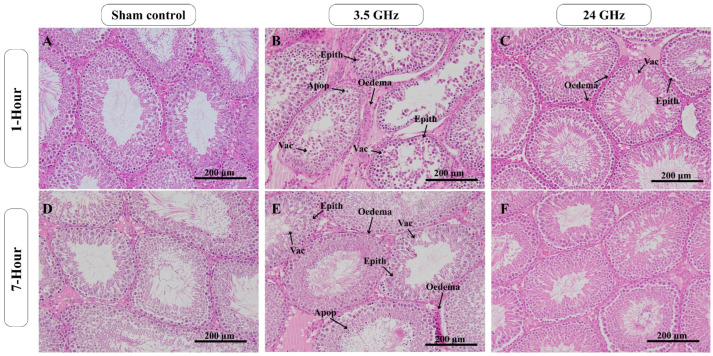
Representative histological photomicrographs of rat testes after 60 days of RF-EMF exposure. Sections were stained with H&E and examined using a 20× objective; scale bar = 200 µm. Panels (**A**–**C**) show animals exposed for 1 h/day: (**A**) sham control, (**B**) 3.5 GHz, and (**C**) 24 GHz. Panels (**D**–**F**) show animals exposed for 7 h/day: (**D**) sham control, (**E**) 3.5 GHz, and (**F**) 24 GHz. Arrows indicate mild lesions identified during blinded analysis: epithelial disorganization (Epith), vacuolation (Vac), apoptotic or pyknotic germ cells (Apop), and mild interstitial edema (Edema).

Representative histological findings are shown in Figure 1. Across all groups, seminiferous tubules generally retained normal architecture, with intact basement membranes, a complete spermatogenic series, and lumina containing spermatozoa. Sham control groups at both exposure durations showed only occasional, minimal clear spaces.

In the 3.5 GHz 1 h/day group, most seminiferous tubules were comparable to controls, although some fields showed mild epithelial irregularity, focal sloughing, scattered cytoplasmic vacuoles, occasional apoptotic or pyknotic germ cells, and mild interstitial edema. A broadly similar pattern was observed in the 3.5 GHz 7 h/day group, in which limited foci of epithelial disorganization, vacuolation, and occasional apoptotic nuclei were present, while most tubules remained unremarkable.

In the 24 GHz 1 h/day group, seminiferous tubules also retained overall architecture, with occasional mild epithelial irregularity, cytoplasmic vacuoles, scattered apoptotic or pyknotic germ cells, and mild interstitial edema. In the 24 GHz 7 h/day group, histological appearance was broadly comparable to sham controls, with only rare focal lesions at the level of individual tubules.

### 2.3. HSP27 Protein Expression

**Figure 2:**
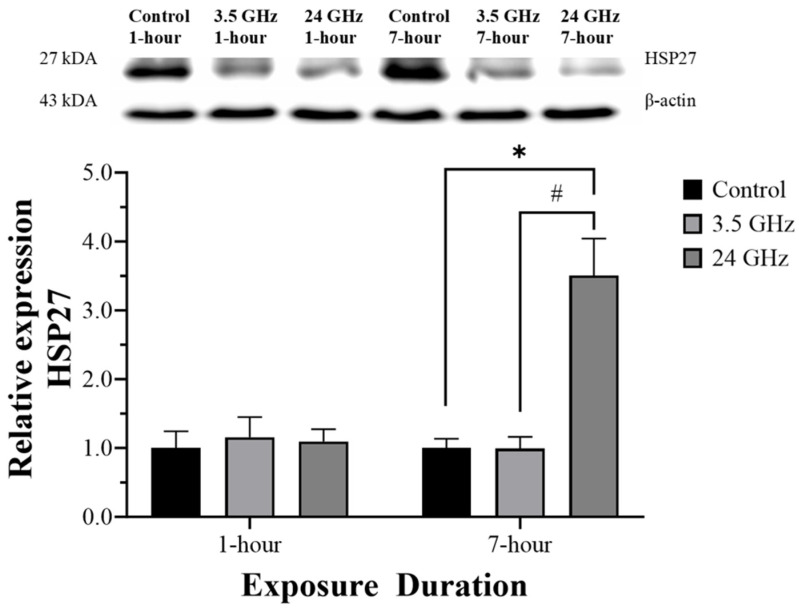
Relative HSP27 protein expression in the control group and groups exposed to 3.5 GHz and 24 GHz for 1 h/day and 7 h/day (n = 6). Data are presented as mean ± SEM. * *p* < 0.05 versus control group. # *p* < 0.05 versus 3.5 GHz group. Original uncropped blot images are provided in Supplementary Figure S1.

Relative HSP27 protein expression is shown in Figure 2. After 1 h/day of exposure, HSP27 expression did not differ significantly among the control, 3.5 GHz, and 24 GHz groups. After 7 h/day of exposure, HSP27 expression was significantly higher in the 24 GHz group (3.51 ± 0.53) than in the control group (1.00 ± 0.13, *p* < 0.05) and the 3.5 GHz group (0.99 ± 0.17, *p* < 0.05).

### 2.4. HSP90 Protein Expression

**Figure 3:**
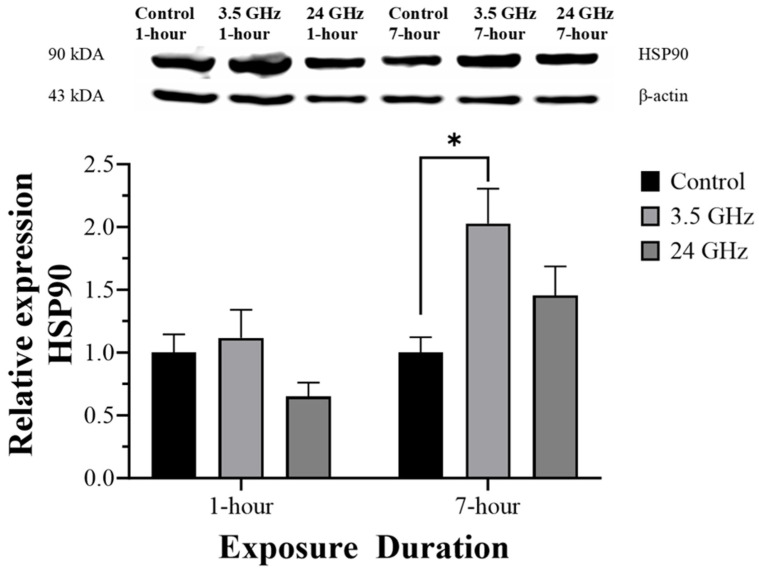
Relative HSP90 protein expression in the control group and groups exposed to 3.5 GHz and 24 GHz for 1 h/day and 7 h/day (n = 6). Data are presented as mean ± SEM. * *p* < 0.05 versus control group. Original uncropped blot images are provided in Supplementary Figure S2.

Relative HSP90 protein expression is shown in Figure 3. After 1 h/day of exposure, HSP90 expression did not differ significantly among groups. After 7 h/day of exposure, HSP90 expression was significantly higher in the 3.5 GHz group (2.03 ± 0.28) than in the control group (1.00 ± 0.12, *p* < 0.05). No other pairwise differences were detected.

### 2.5. HSP70 Protein Expression

**Figure 4:**
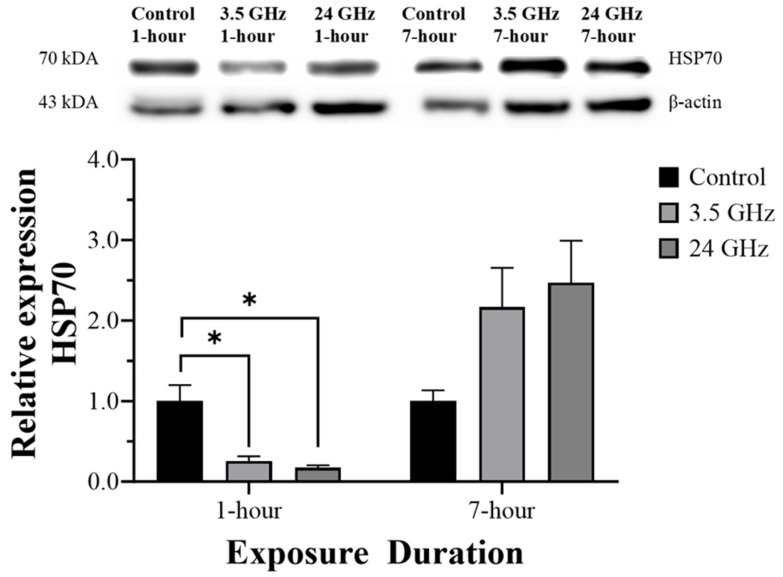
Relative HSP70 protein expression in the control group and groups exposed to 3.5 GHz and 24 GHz for 1 h/day and 7 h/day (n = 6). Data are presented as mean ± SEM. * *p* < 0.05 versus control group. Original uncropped blot images are provided in Supplementary Figure S3.

Relative HSP70 protein expression is shown in Figure 4. After 1 h/day of exposure, HSP70 expression was significantly lower in the 3.5 GHz group (0.25 ± 0.06) and the 24 GHz group (0.17 ± 0.03) than in the control group (1.00 ± 0.20, *p* < 0.05). After 7 h/day of exposure, HSP70 expression did not differ significantly among groups.

## 3. Discussion

Interpretation of RF-EMF bioeffects depends fundamentally on dosimetry because the established adverse effects that underpin current exposure guidelines are primarily thermal in nature. In the present study, numerical dosimetry indicated a low-exposure regime at the 20 cm source-to-animal distance with higher estimated whole-body and local SAR at 3.5 GHz than at 24 GHz. At 3.5 GHz, the extrapolated local SAR and total SAR remained below the general public basic restrictions in the ICNIRP guidelines and the corresponding unrestricted-environment dosimetric reference limits in IEEE C95.1-2019 [2,15]. However, local exposure at 24 GHz is more appropriately interpreted using absorbed power density rather than local SAR because above 6 GHz, energy deposition becomes increasingly superficial [2]. Accordingly, the 24 GHz SAR values in the present work are best regarded as comparative descriptors of volumetric absorption that are useful for cross-frequency interpretation rather than as the primary compliance metric.

Histologically, seminiferous tubule architecture was largely preserved across groups with only minimal focal epithelial irregularity, vacuolation, occasional apoptotic or pyknotic germ cells, and mild interstitial edema. No pattern of advanced or widespread degeneration was observed. This finding is consistent with the broader studies on experimental RF-EMF-related effects on male reproduction, where they are often more evident in molecular or sperm-related endpoints than in changes in dose-dependent testicular histopathology. Recent systematic evaluation of experimental studies similarly concluded that RF-EMF may affect testicular tissue and sperm quality, but that the evidence remains uncertain, and surrogate changes do not necessarily translate into functional reproductive impairment [16].

Despite the limited structural alteration, testicular heat shock proteins were modulated in a frequency- and duration-dependent manner, supporting the view that molecular stress markers may shift even when overt tissue injury is not apparent. After 7 h/day of exposure, HSP27 was selectively increased in the 24 GHz group. This is biologically plausible because HSP27 is a stress-responsive small heat shock protein involved in actin cytoskeletal regulation, redox homeostasis, and cytoprotection [17,18]. In the testis, HSP27 is expressed in specific somatic and germ cell populations, and altered expression has been associated with abnormal spermatogenesis [19]. Given that mmWave absorption is expected to be substantially more superficial than mid-band absorption, the 24 GHz pattern observed here may reflect a distinct stress-adaptation response associated with superficial energy deposition rather than broad volumetric interaction with deeper tissue. However, the present data do not permit firm conclusions regarding the precise pathway linking exposure to intratesticular HSP27 modulation.

In contrast, 3.5 GHz exposure was associated with a selective increase in HSP90 after 7 h/day. Relative to 24 GHz, mid-band frequencies are expected to produce deeper and more spatially distributed absorption, which is consistent with the higher estimated SAR values observed at 3.5 GHz in the present dosimetry. HSP90 is a major molecular chaperone involved in the stabilization and functional regulation of client proteins, including proteins relevant to spermatogenesis and male fertility [10,12]. In this context, the delayed HSP90 increase after repeated 3.5 GHz exposure is compatible with activation of proteostatic adaptation under sustained low-level stress, rather than with overt tissue injury. This interpretation is also consistent with the preserved seminiferous histoarchitecture observed in the exposed groups.

A notable finding was the significant reduction in HSP70 expression in both the 3.5 GHz and 24 GHz groups after 1 h/day exposure, whereas no between-group differences were detected after 7 h/day exposure. This early downregulation contrasts with the classical heat shock response, in which acute cellular stress often activates heat shock factors and induces chaperone expression [20]. Because HSP70 family members are closely linked to germ-cell development and meiotic progression, altered HSP70 regulation in the testis may be biologically meaningful even in the absence of overt histological injury [12,21]. Nevertheless, the present study does not distinguish whether this early reduction reflects transient suppression of chaperone signaling, a rapid adaptive feedback event, or other upstream regulatory changes. The absence of a persistent difference after prolonged daily exposure suggests that the early response may have normalized over time or remained below the threshold for sustained induction.

Taken together, the divergent temporal profiles of HSP27, HSP70, and HSP90 suggest that low-level 5G-relevant RF-EMF can modulate testicular stress signaling in a frequency- and duration-dependent manner. Within this framework, 3.5 GHz was associated with a later HSP90 response, whereas 24 GHz was associated with a later HSP27 response, while both frequencies were associated with early HSP70 suppression. These findings are consistent with the concept that frequency-dependent dosimetric characteristics may influence not only the magnitude but also the pattern of molecular adaptation in reproductive tissue. At the same time, because histological injury was minimal, the present data are more supportive of subtle stress-response remodeling than of testicular toxicity. Whether these HSP changes represent adaptive protection or early adverse signaling cannot be resolved without integration with reproductive functional endpoints and more detailed mechanistic assays.

Several limitations should be considered. First, the 20 cm SAR values were estimated by local power-law extrapolation from simulations performed at 5 cm and 10 cm rather than measured directly in a cage or simulated at the full exposure distance. These values should therefore be interpreted as approximate dosimetric estimates. Second, the rats were unrestrained, so posture and position likely introduced variation in local field distribution. Third, for the 24 GHz exposure, absorbed power density and direct temperature monitoring were not assessed in the present study. Because these parameters are particularly relevant for higher-frequency near-field exposure assessment, a thermal contribution could not be directly excluded, and interpretation should therefore remain cautious [1,2]. Finally, histology and HSP expression were assessed only at the end of the 60-day exposure period without intermediate or recovery time points. In addition, the present study focused on testicular histological and molecular endpoints and did not include systemic blood-based biomarkers or gross examination of major organs. Future studies should therefore combine refined in-cage dosimetry, position-sensitive exposure characterization, temperature assessment, and additional functional reproductive endpoints. Exposure scenarios involving closer source placement, including configurations relevant to personal device carriage, would also be informative but should be evaluated as distinct dosimetric conditions rather than direct extensions of the present geometry.

In summary, under low-level exposure conditions defined by numerical dosimetry at a 20 cm source-to-animal distance, repeated 3.5 GHz and 24 GHz RF-EMF exposure preserved overall seminiferous tubule structure but altered testicular HSP27, HSP70, and HSP90 expression in a frequency- and duration-dependent manner. The present data support frequency- and duration-associated modulation of testicular heat shock protein expression under the tested exposure conditions, but the extent to which these changes were independent of thermal contributions remains to be clarified in future studies incorporating direct temperature assessment.

## 4. Materials and Methods

**Figure 5:**
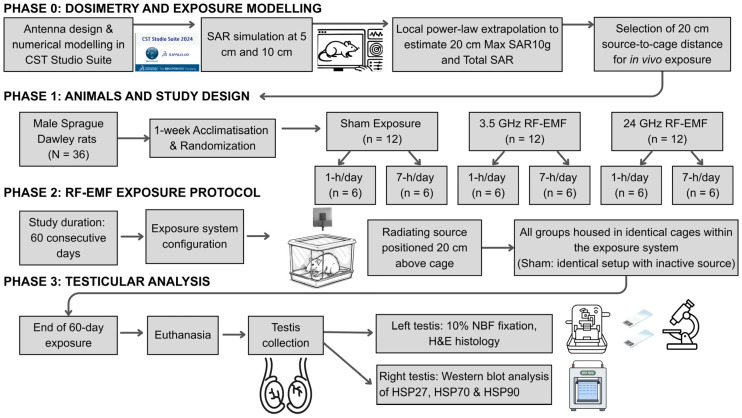
Schematic overview of the dosimetry workflow, animal grouping, RF-EMF exposure protocol, and testicular endpoints.

A schematic overview of the study design, including numerical dosimetry, animal grouping, RF-EMF exposure protocol, and testicular endpoints, is shown in Figure 5.

### 4.1. Antenna Design and Numerical Dosimetry

Two microstrip antennas were designed by UTeM and tuned to 3.5 GHz and 24 GHz and were modeled in CST Studio Suite 2024 (Dassault Systèmes, Vélizy-Villacoublay, France). For each frequency and separation distance, the antenna was excited using the CST port definition, and the accepted power at the feed port (P_acc_) was recorded. Raw SAR outputs were normalized to 1 W accepted power by dividing the simulated SAR values by Pacc. The normalized maximum SAR averaged over 10 g tissue (Max SAR_10g_) and whole-body average SAR (Total SAR) were obtained using the CST post-processing tools.

The antenna model was positioned directly above an adult male rat voxel model at source-to-model separations of 5 cm and 10 cm. For each frequency-distance combination, normalized Max SAR_10g_ and Total SAR were computed. To estimate the SAR values corresponding to the 20 cm antenna-to-cage distance used in the biological experiment, a local power-law extrapolation was applied:

$$S\left(r\right)=S_{10}{\left(\frac{10}{r}\right)}^{n}$$

where *S*(r) is the SAR metric at source-to-animal distance r, *S*_10_ is the normalized SAR value at 10 cm, and n is the local fall-off exponent derived from the simulated 5 cm and 10 cm values:

$$n=\frac{\ln\left(S_{5}/S_{10}\right)}{\ln\left(10/5\right)}$$

This extrapolation was applied separately to Max SAR_10g_ and Total SAR for each frequency to estimate the corresponding values at 20 cm. It served as a local approximation to obtain order-of-magnitude estimates for the 20 cm exposure distance. It should not be interpreted as a fully validated distance-scaling model for the entire near-field geometry.

### 4.2. Animals and Experimental Design

Thirty-six adult male Sprague Dawley rats aged 8 weeks and weighing 200–250 g were obtained from the Laboratory Animal Resource Unit, Faculty of Medicine, Universiti Kebangsaan Malaysia. Animals were housed individually in ventilated cages under a 12 h light-dark cycle at 25 ± 1 °C, with ad libitum access to food and water, and were acclimatized for 1 week before the experiment.

The rats were randomly assigned to three groups: sham control, 3.5 GHz, and 24 GHz. Within each group, animals were further allocated to 1 h/day or 7 h/day exposure for 60 consecutive days, giving six animals per subgroup.

All procedures involving animal handling and care complied with the guidelines of the UKM Animal Ethics Committee and were approved under ethical approval code FP/2023/FARAH HANAN/15-FEB./1310-FEB.-2023-FEB.-2025.

### 4.3. RF-EMF Exposure Setup

The 3.5 GHz antenna design was fabricated by UTeM and used as the transmit antenna for the 3.5 GHz RF-EMF exposure. A commercial HLK-LD2420 module (Hi-Link, Shenzhen, China) was used as the transmit antenna for the 24 GHz RF-EMF exposure. For the 3.5 GHz groups, exposure was generated using a SynthHD (v2) dual-channel microwave generator covering 10 MHz to 15 GHz (Windfreak Technologies, New Port Richey, FL, USA). For the 24 GHz groups, exposure was generated using a commercially available human presence detector integrated with the HLK-LD2420 millimeter-wave antenna module.

In both setups, the radiating source was mounted 20 cm above the rat cages. This distance was selected to standardize the exposure geometry across frequencies and to reduce near-field variability. The selected spacing was also consistent with previous studies [22,23].

For the 3.5 GHz system, the transmitted field was monitored continuously in real time using a USB-SA124B 12.4 GHz spectrum analyzer (Signal Hound, Battle Ground, WA, USA) running Spike software (version 3.12), which provided live visualization and scheduled recordings to verify carrier stability and device uptime throughout each exposure session. For the 24 GHz system, transmitter output from the HLK-LD2420 module was checked before and after each exposure using a Keysight N9010A EXA signal analyzer (Keysight Technologies, Santa Rosa, CA, USA) to verify emission within the expected 24–24.25 GHz band before and after each exposure session. The device companion application was also used to monitor operational status during the exposure period. Representative analyzer outputs used to verify the emitted frequencies of the 3.5 GHz and 24 GHz exposure systems are provided in Supplementary Figures S4 and S5, respectively.

During exposure, rats were housed individually in non-metallic cages arranged in a carousel configuration to promote relatively uniform RF-EMF field distribution and consistent whole-body exposure across all animals. The animals were not physically restrained and were free to move within their enclosures throughout the exposure period. All metallic components within the cages were removed during exposure and replaced with plastic food and water containers to minimize reflection and secondary absorption of RF-EMF. Sham control rats for the 1 h/day and 7 h/day cohorts were housed in an identical carousel arrangement at the same distance from an inactive antenna and were handled in the same manner as the exposed groups. A representative photograph of the actual experimental setup is provided in Supplementary Figure S6. All procedures were carried out under standard laboratory conditions to minimize non-specific physiological stress unrelated to RF-EMF exposure.

### 4.4. Euthanasia and Testes Collection

At the end of the exposure period, rats were euthanized under deep anesthesia induced by intraperitoneal injection of a ketamine-xylazine-tiletamine-zolazepam working solution at 1.0 mL/kg body weight. The working solution contained ketamine from Ilium Ketamil Injection and xylazine from Ilium Xylazil-100 (Troy Laboratories Pty Ltd., Glendenning, NSW, Australia), and tiletamine-zolazepam from Zoletil-100 (Virbac, Carros, France), providing final working concentrations of ketamine (6.67 mg/mL), xylazine (6.67 mg/mL), tiletamine (3.33 mg/mL), and zolazepam (3.33 mg/mL). Adequate anesthesia was confirmed by loss of righting, tail-pinch, pedal, and corneal reflexes. The testes were then excised and weighed. The left testis was fixed in 10% neutral buffered formalin (NBF) for histological analysis, whereas the right testis was snap-frozen and stored at −80 °C for protein analysis.

### 4.5. Histological Evaluation of the Testis

Fixed testes were processed routinely, paraffin-embedded, sectioned at 5 µm, and stained with hematoxylin and eosin. Slides were examined by blinded readers using randomized codes on a light microscope (Olympus BX53F, Olympus Corporation, Hachioji, Japan) at 20× magnification with a 200 µm scale bar. For each animal, 10 complete and near-round seminiferous tubules with intact basement membranes were evaluated.

Histological features were assessed qualitatively for overall testicular architecture, tubule organization, and germ cell integrity. Epithelial disorganization or sloughing, cytoplasmic vacuolation, apoptotic or pyknotic nuclei, multinucleated germ cells, and interstitial changes, including edema, were categorized according to distribution and apparent severity. Findings are reported descriptively with representative photomicrographs.

### 4.6. Measurement of HSP Expression by Western Blotting

Western blotting was used to assess testicular HSP27, HSP70, and HSP90 expression. Approximately 0.3 g of testicular tissue was homogenized in RIPA Lysis Buffer (Strong) supplemented with PMSF and Na_3_VO_4_ (Elabscience, Wuhan, China; Cat. No. E-BC-R327), then centrifuged at 12,000× *g* for 20 min at 4 °C. Protein concentration was measured using the Pierce™ BCA Protein Assay Kit (Thermo Fisher Scientific, Waltham, MA, USA; Cat. No. 23227).

Equal amounts of protein (50 µg) were separated by SDS-PAGE using an SDS-PAGE Gel Kit (Elabscience, China; Cat. No. E-IR-R305), with 10% gels used for HSP70 and HSP90 and 15% gels for HSP27, and then transferred to PVDF membranes. Membranes were blocked with 5% skim milk in TBST for 1 h and incubated overnight at 4 °C with primary antibodies against HSP90 alpha (rabbit polyclonal, 1:1000; Elabscience, China; Cat. No. E-AB-70141), HSP70 (rabbit polyclonal, 1:1000; Elabscience, China; Cat. No. E-AB-40490), HSP27 (rabbit polyclonal, 1:500; Elabscience, China; Cat. No. E-AB-31748), and β-actin (HRP-conjugated, 1:1000; Abcam, Cambridge, UK; Cat. No. ab49900). After washing in TBST, membranes for HSP90, HSP70, and HSP27 were incubated with goat anti-rabbit IgG (H + L), HRP-conjugated secondary antibody (1:5000; Elabscience, China; Cat. No. E-AB-1003) for 1 h at room temperature. Bands were developed using the Excellent Chemiluminescent Substrate (ECL) Kit (Elabscience, China; Cat. No. E-IR-R301), imaged using an Amersham Imager 600 (Cytiva, Marlborough, MA, USA), and quantified by densitometry using Image Lab software version 6.0.1 (Bio-Rad Laboratories, Hercules, CA, USA). Target protein expression was normalized to β-actin. Original uncropped Western blot images corresponding to HSP27, HSP90, HSP70, and their respective β-actin controls are provided in Supplementary Figures S1–S3.

### 4.7. Statistical Analysis

Statistical analyses were performed using GraphPad Prism version 10 (GraphPad Software, Boston, MA, USA). Normality was assessed using the Shapiro–Wilk test. Within each exposure duration (1 h/day and 7 h/day), group differences in relative HSP expression were analyzed using one-way ANOVA followed by Tukey’s post hoc test. Data are presented as mean ± SEM, and *p* < 0.05 was considered statistically significant.

## 5. Conclusions

Although the microscopic structure of the seminiferous tubules was primarily preserved during H&E evaluation, prolonged exposure to 3.5 GHz and 24 GHz RF-EMF still caused frequency- and time-dependent changes in the testicular heat shock protein expression of male Sprague Dawley rats. While dosimetry models indicated low overall exposure, it should be noted that the 20 cm in vivo values were estimates based on 5 cm and 10 cm simulations. Together, these findings support the use of HSP27, HSP70, and HSP90 as sensitive molecular indicators of testicular stress under 5G-relevant RF-EMF exposure conditions. However, further studies incorporating direct temperature assessment and refined dosimetry are needed to clarify the underlying mechanisms.

## Figures and Tables

**Table 1:** Max SAR_10g_ in W kg^−1^ in the rat voxel model at 3.5 GHz and 24 GHz for antenna–model separations of 5 and 10 cm (simulated) and 20 cm (extrapolated).

Distance (cm)	3.5 GHz (W kg^−1^)	24 GHz (W kg^−1^)
5	0.95	2.15
10	0.38	9.30 × 10^−4^
20	0.15	4.00 × 10^−7^

**Table 2:** Total SAR in W kg^−1^ in the rat voxel model at 3.5 GHz and 24 GHz for antenna–model separations of 5 and 10 cm (simulated) and 20 cm (extrapolated).

Distance (cm)	3.5 GHz (W kg^−1^)	24 GHz (W kg^−1^)
5	0.22	0.22
10	0.12	1.97 × 10^−4^
20	0.06	1.80 × 10^−7^

## Data Availability

The datasets generated and analyzed during the current study are available from the corresponding author upon reasonable request.

## Supplementary Materials

The following supporting information can be downloaded at https://www.mdpi.com/article/10.3390/ijms27083452/s1.

## Abbreviations

The following abbreviations are used in this manuscript:

5G	Fifth generation
HSP	Heat shock protein
Max SAR_10g_	Maximum specific absorption rate averaged over 10 g tissue
mmWave	Millimeter wave
P_acc_	Accepted power at the feed port
RF-EMF	Radiofrequency electromagnetic field
SAR	Specific absorption rate
Total SAR	Whole-body average specific absorption rate
SEM	Standard error of the mean
UTeM	Universiti Teknikal Malaysia Melaka
UKM	Universiti Kebangsaan Malaysia
MOHE	Ministry of Higher Education
FRGS	Fundamental Research Grant Scheme
IACUC	Institutional Animal Care and Use Committee
CST	Computer simulation technology
ICNIRP	International Commission on Non-Ionizing Radiation Protection
IEEE	Institute of Electrical and Electronics Engineers
NBF	Neutral buffered formalin
H&E	Hematoxylin and eosin
SDS-PAGE	Sodium dodecyl sulfate-polyacrylamide gel electrophoresis
PVDF	Polyvinylidene fluoride
TBST	Tris-buffered saline with Tween 20
HRP	Horseradish peroxidase
ECL	Excellent chemiluminescent substrate
RIPA	Radioimmunoprecipitation assay
PMSF	Phenylmethylsulfonyl fluoride
BCA	Bicinchoninic acid
