# Supplementary figures and images for: Testicular Heat-Shock Protein Expression in Rats Following 3.5 GHz and 24 GHz RF-EMF Exposure
Source: Int J Mol Sci. 2026 Apr 12;27(8):3452. doi: 10.3390/ijms27083452 (PMC13115579; doi:10.3390/ijms27083452)

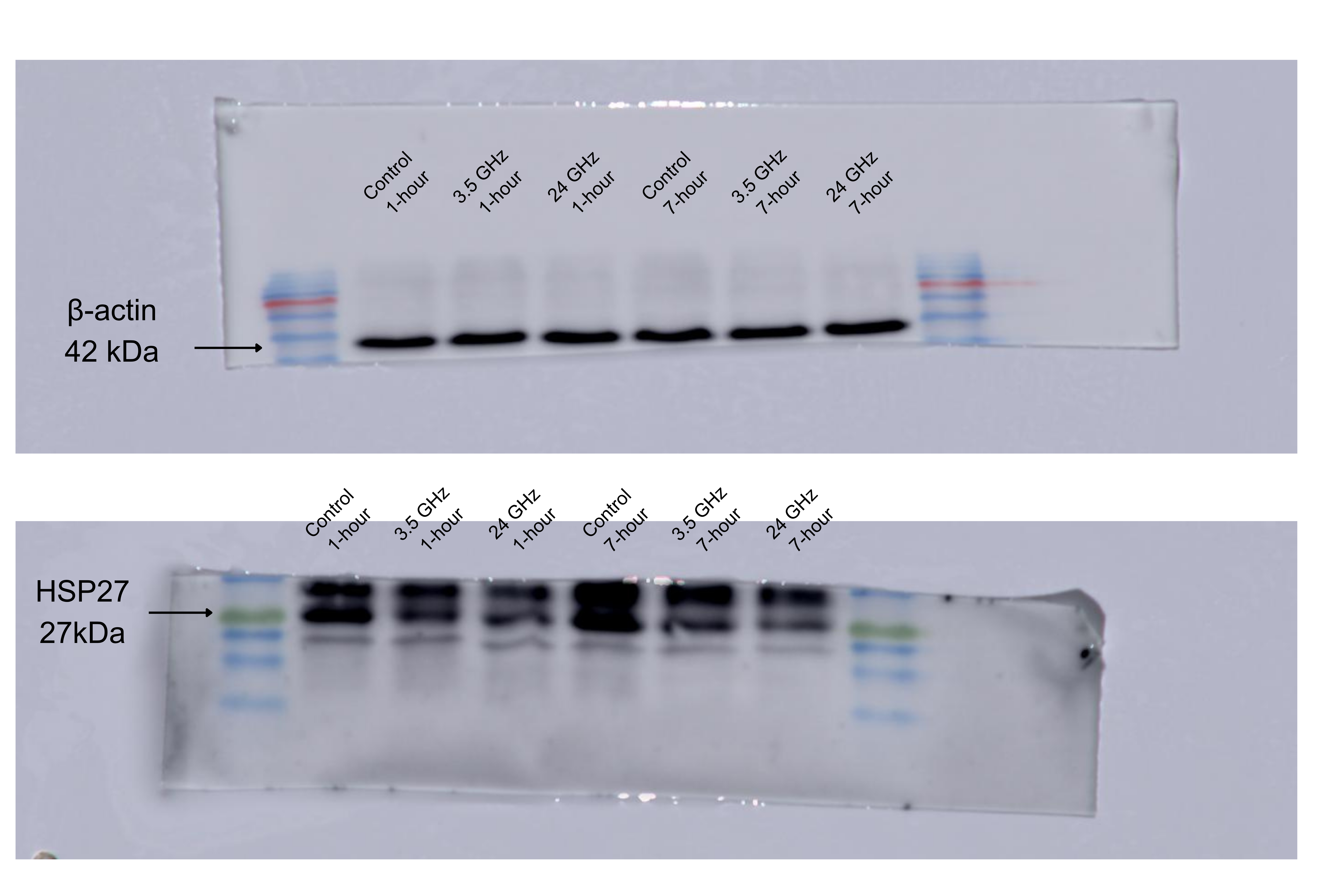

Supplement: Supplementary file 1 [file ijms-27-03452-s001.zip › Supplementary Figure S1 uncropped HSP27.png]

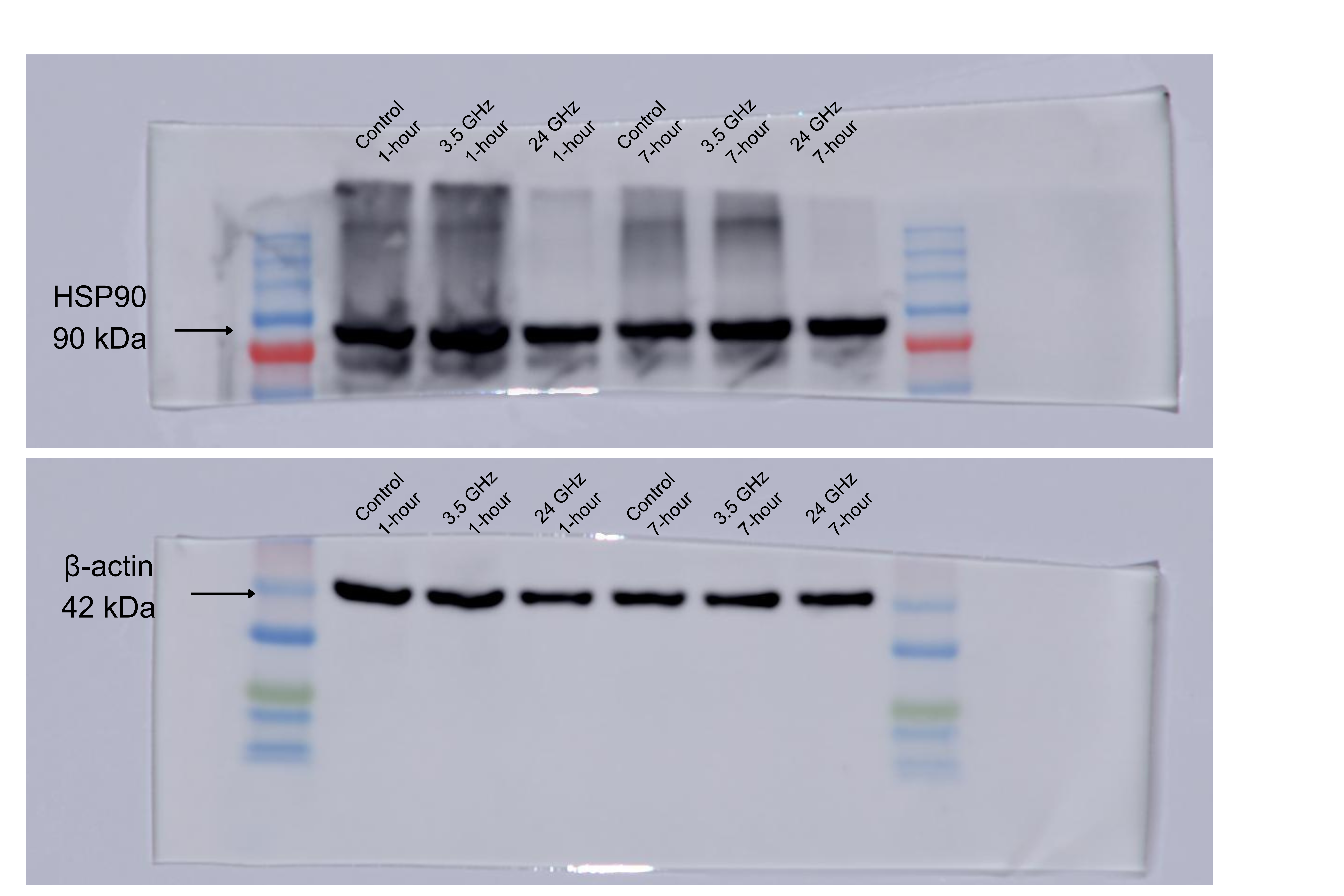

Supplement: Supplementary file 1 [file ijms-27-03452-s001.zip › Supplementary Figure S2 uncropped HSP90.png]

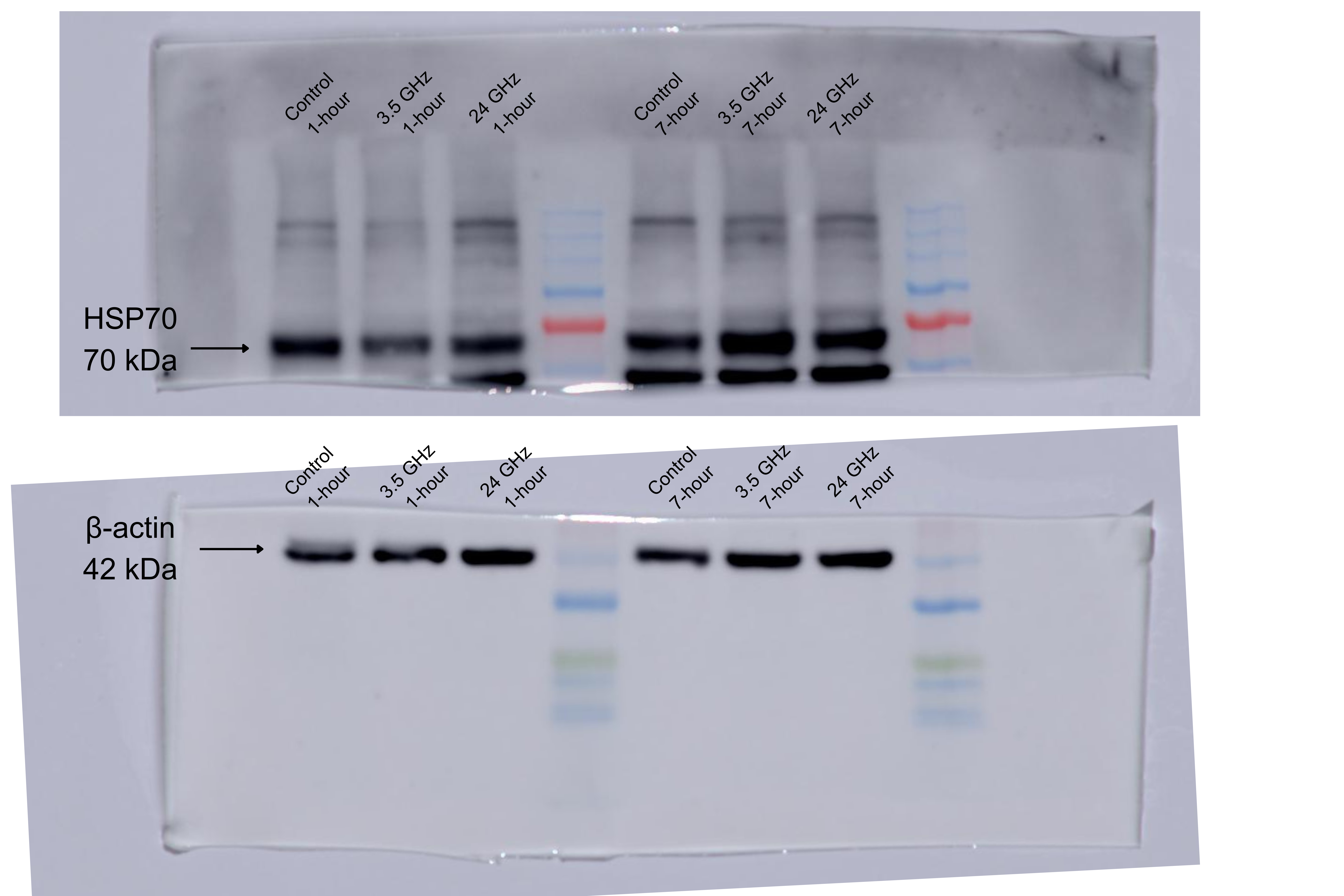

Supplement: Supplementary file 1 [file ijms-27-03452-s001.zip › Supplementary Figure S3 uncropped HSP70.png]

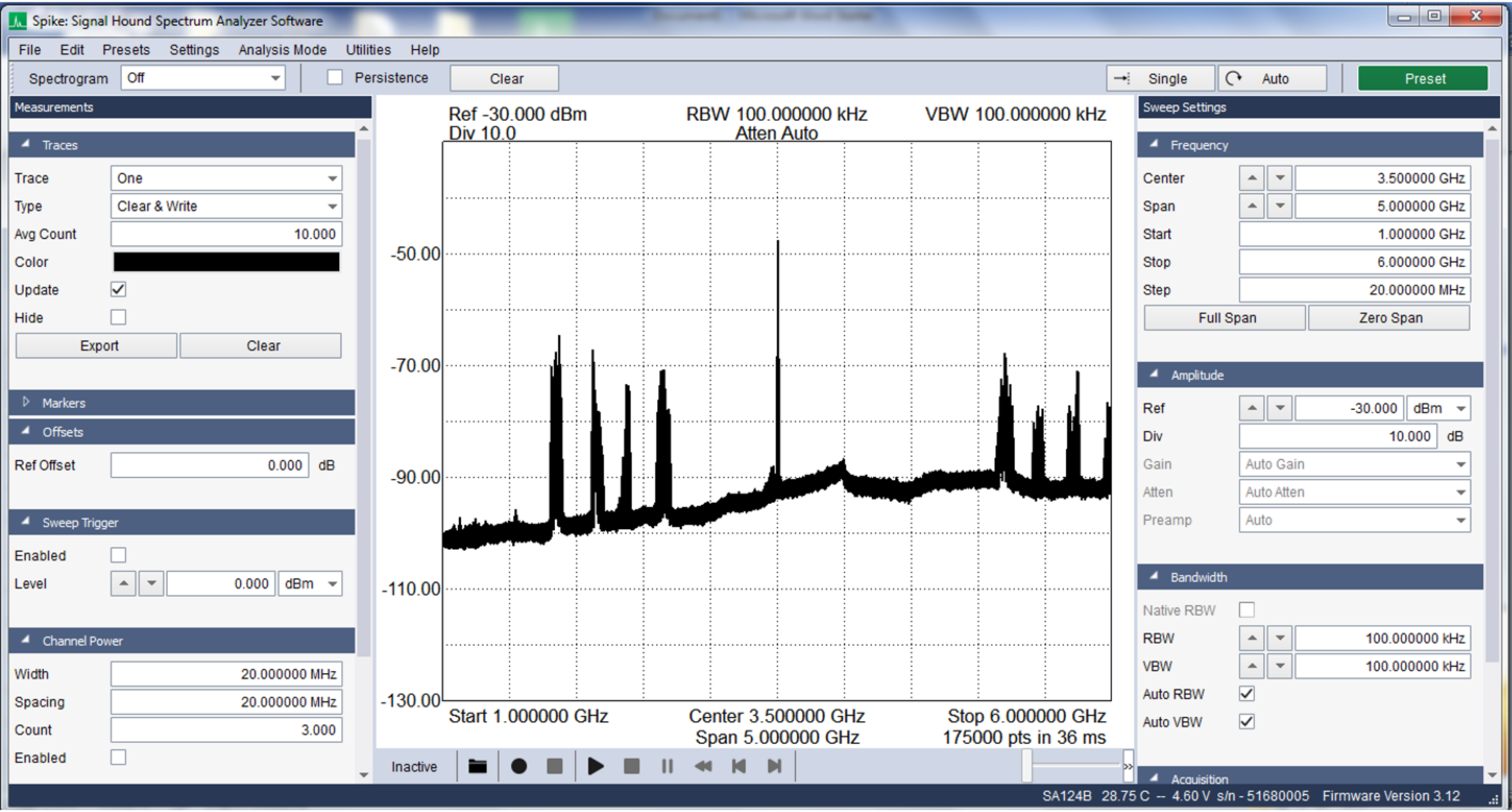

Supplement: Supplementary file 1 [file ijms-27-03452-s001.zip › Supplementary Figure S4 3.5 GHz analyzer output.png]

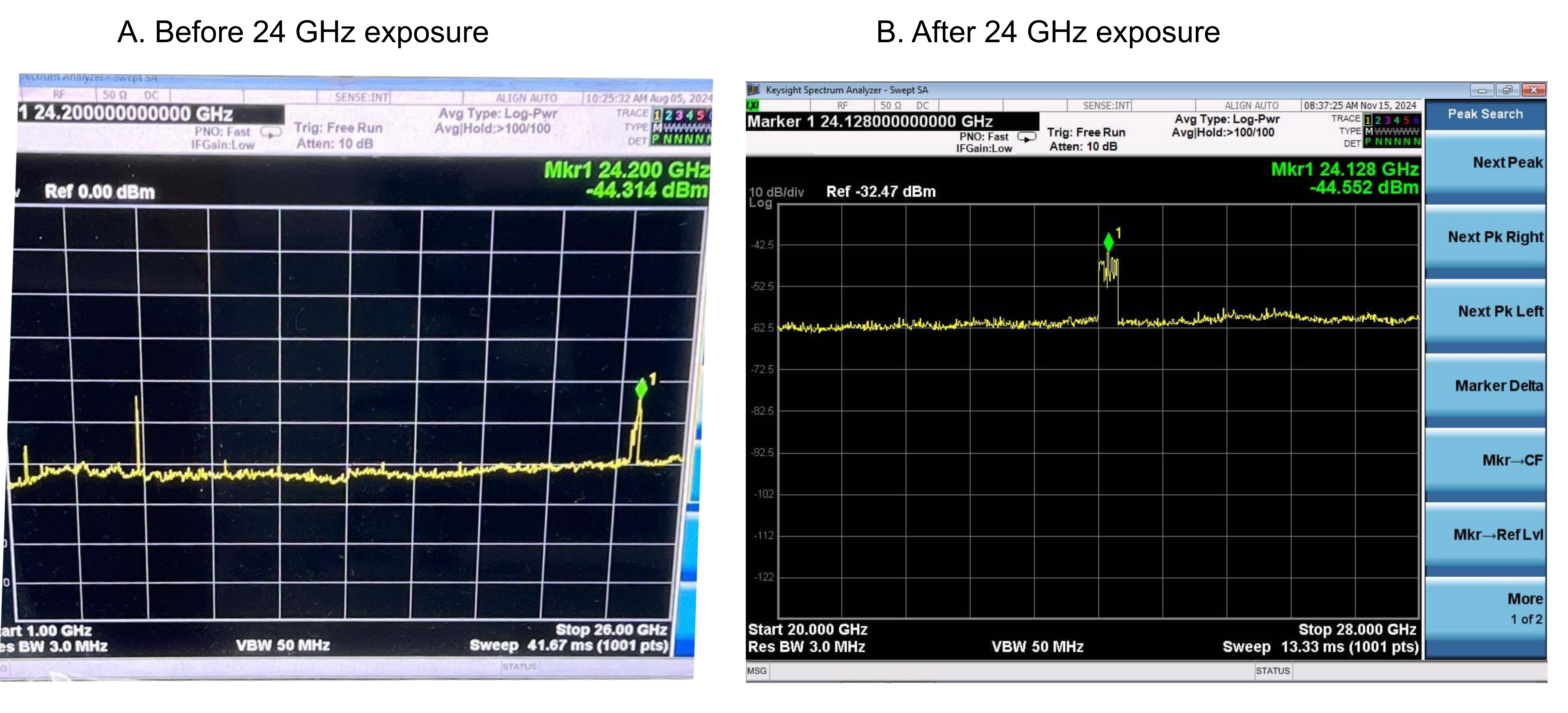

Supplement: Supplementary file 1 [file ijms-27-03452-s001.zip › Supplementary Figure S5 24 GHz analyzer output.png]

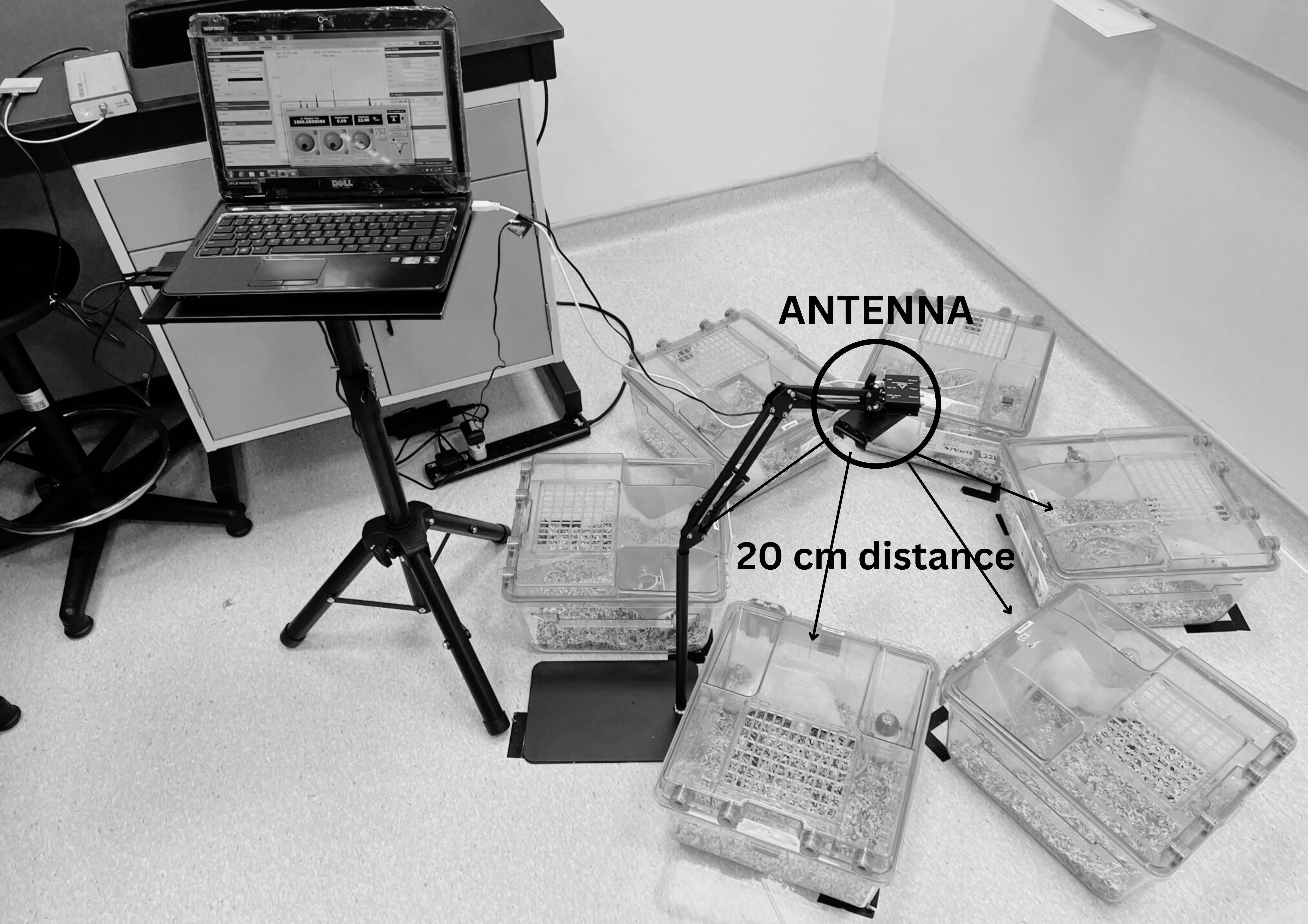

Supplement: Supplementary file 1 [file ijms-27-03452-s001.zip › Supplementary Figure S6 exposure experimental setup.jpg]
